# The Diterpene Sclareol Vascular Effect in Normotensive and Hypertensive Rats

**DOI:** 10.5935/abc.20170086

**Published:** 2017-08

**Authors:** Debora Ribeiro Campos, Andrea Carla Celotto, Agnes Afrodite S. Albuquerque, Luciana Garros Ferreira, Ariadne Santana e Neves Monteiro, Eduardo Barbosa Coelho, Paulo Roberto Barbosa Evora

**Affiliations:** Universidade de São Paulo, São Paulo, SP - Brazil

**Keywords:** Diterpenes / therapeutic use, Rats, Hypertension, Anti-Infective Agents, Cytotoxins

## Abstract

**Background:**

The diterpene Sclareol has antimicrobial action, cytotoxic and cytostatic effects and anti-tumor activities. However, researches on the cardiovascular system are scarce.

**Objective:**

This study was designed to investigate the mechanisms involved in the Sclareol cardiovascular effect in normotensive and hypertensive rats.

**Methods:**

The arterial hypertension was promoted using 2-kidneys 1-clip model in rats. The effect of sclareol on blood pressure was performed by using three dose (10, 20 and 40 mg/kg). Cumulative dose-response curves for Sclareol were determined for endothelium-intact and endothelium-denuded aortic rings in presence or absence of L-NAME and ODQ. The NOx levels were measure in the plasma sample.

**Results:**

The Sclareol administration in vivo caused a significant reduction in blood pressure in both groups. In vitro the sclareol promoted relaxation in aorta, with endothelium, pre-contracted to Phe. The inhibitors of the nitric oxide synthase and soluble guanylate cyclase were as efficient as the removal of endothelium, in inhibiting the Sclareol-induced relaxation. Otherwise, it was no change of NOx. Also, for unknown reasons, the Sclareol is not selective for hypertensive animals.

**Conclusion:**

1. The diterpene Sclareol showed in vivo hypotensive and in-vitro vasodilator effects;
2. The chemiluminescence plasmatic NO analysis showed no significant difference between groups and
3. The Sclareol exhibit better effect on normotensive than hypertensive animals to reduce blood pressure. It is concluded that the diterpenes metabolites would be a promising source prototype for the development of new agents in the cardiovascular therapy.

## Introduction

The plant kingdom has contributed in a significant way to provide substances useful in the treatment of diseases that affect humans. In this context, diterpenes are a large class of secondary metabolites produced by plants and have many important biological activities.^1^ Several studies sighted these substances as a promising source of new leads for the discovery and development of new agents for use in cardiovascular therapy, and have shown that many diterpenoid classes exert the significant effect on the cardiovascular system.^2-5^ These studies suggest that metabolites class as a promising source prototype for the development of new agents in the cardiovascular therapy. The diterpenes are synthesized in plants located in plastids, but can also be synthesized by certain insects and marine organisms.

**Figure 1:**
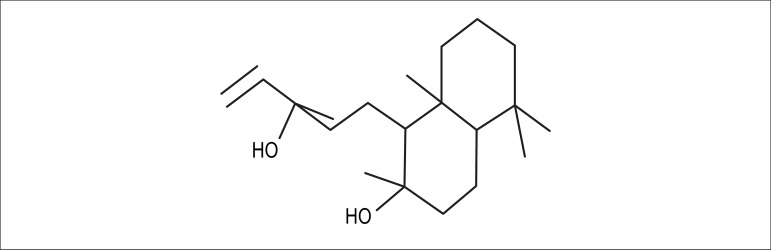
Sclareol molecular structure.

The diterpene Sclareol (Figure 1) is extracted from inflorescences Salviasclarea L., relatively easy to grow grass and high throughput.^6^ Studies using this compound showed its antimicrobial action, cytotoxic and cytostatic effects on leukemic cell lines and anti-tumor activities.^7-10^ However, studies about this compound on the cardiovascular system are scarce, or maybe have never been studied. So it is crucial that such investigations are carried out, considering that this compound is highly available and secure for testing. Therefore, this study was designed to investigate the mechanisms involved in cardiovascular effect (in vitro and in vivo) of diterpene Sclareol in normotensive and hypertensive rats.

## Methods

### Ethics statement and animals

The experimental procedures and policies for animal handling were reviewed and approved by the Institutional Committee for Animal Care and Use of the School of Medicine of Ribeirão Preto, the University of São Paulo, and were by the European Commission's Directive 2010/63/EU. Twenty male Wistar rats (180-220 g) were housed under standard laboratory conditions (12 h light/dark cycle at 21°C) with free access to food and water. The animals were randomly by lot and divided into two groups of 7 animals: normotensive and hypertensive for blood pressure protocols and 6 control animals for vascular reactivity protocol. The rats of the hypertensive group underwent the surgical procedure 2K1C for hypertension induction while the animals of the normotensive group were sham-operated.

### Drugs

Acetylcholine (ACh), 1H-[1,2,4] oxadiazole [4,3-a]quinoxalin-1-one (ODQ), phenylephrine (Phe) and Sclareol were purchased from Sigma Chemical Company (St. Louis, MO, USA). Nω-nitro-L-arginine methyl ester (L-NAME) was obtained from Calbiochem (San Diego, CA, USA). Isoflurane from Abbott. All the salts used for Krebs solution preparation were furnished by Vetec Química Fina Ltda. Almost all the drugs were prepared with distilled water, except for indomethacin (which was dissolved in ethanol) and Sclareol (solubilized in dimethyl sulfoxide and diluted in ethanol + water).

### Induction of the hypertension

The animals were anesthetized with ketamine (50 mg/kg) and xylazine (10 mg/kg) intraperitoneally. After complete anesthesia, the rats were submitted to a laparotomy: half of the animals had partial constriction of the main left renal artery with a silver clip with a 0.10 mm gap (2K1C) (Goldblatt et al.,^11^) and half of them had the main left renal artery isolated but did not receive the clip (sham). In order to monitor the hypertension development, the SBP was non-invasively measured by means of a tail cuff, once a week. (Kent Scientific Corporation, Connecticut, USA).^12^ The 2K1C rats were considered hypertensive if they had tail SBP ≥ 160 mmHg at 3^th^ week after the surgical procedures. The 2K1C rats with SBP < 160 mmHg at 3^th^ week were euthanatized. The sham operated rats constituted the normotensive group. Three weeks after hypertension induction, the animals were anesthetized, and the femoral artery and vein were cannulated for continuous measurement of systolic blood pressure (SBP) and drugs administration, respectively.

### Sclareol effect on the systolic blood pressure

After anesthesia (urethane, 2 g/kg, intraperitoneal), vascular cannulation and stabilization period (20 minutes) with continuous real time SBP recording, three doses of Sclareol (10, 20 and 40 mg/kg) or vehicle (DMSO and water+ethanol) were administered to the normotensive and hypertensive rats. The vehicle administration was performed before sclareol curve in the both groups and because we didn’t have difference between normotensive and hypertensive, we mixed then. Each dose was given in a 200 *µ*L intravenous bolus and the interval between two consecutives doses was 6 minutes (time required for the SBP return to baseline values). For each animal, the variation in systolic blood pressure (ΔSBP) was calculated subtracting the mean of the lowest SBP values immediately after Sclareol administration from the mean of the baseline SBP values before Sclareol bolus. The monitoring of mean arterial blood pressure was measured using MP System 100 A (BioPac System, Inc., Santa Barbara, CA, USA).

### Vascular reactivity

Five male Wistar rats (280-300 g) were anesthetized with inhalational isoflurane, followed by laparotomy for exsanguination via abdominal aorta and thoracotomy for thoracic aorta harvesting. The thoracic aorta was carefully dissected, confirmed to be free of connective tissue, and immediately immersed in Krebs solution. The Krebs solution was composed of NaCl (118.0 mM), KCl (4.7 mM), CaCl2 (2.5 mM), KH2PO4 (1.2 mM), MgSO4 (1.66 mM), glucose (11.1 mM), and NaHCO3 (25.0 mM); the solution had a pH 7.4. The thoracic aorta immersed in Krebs solution was cut into rings that were 4-5 mm in length. The endothelium was removed from some of the rings by gently rubbing the intimal surface of the blood vessel with a pair of watchmaker's forceps. This procedure effectively removes the endothelium, but it does not affect the ability of the vascular smooth muscle to contract or relax. The aortic rings were placed in isolated organ baths (10 mL) filled with Krebs solution, maintained at 37°C, and bubbled with 95% O_2_/5% CO_2_ (pH 7.4). Each arterial ring was suspended by two stainless steel clips that were inserted through the lumen. One clip was anchored to the bottom of the organ bath while the other was connected to a strain gauge to measure the isometric force with the aid of the Grass FT03 equipment (Grass Instrument Company, Quincy, MA, USA). Each ring was stretched to the optimal length-tension of 2.0 g, which had been determined in a pilot study, and was allowed to equilibrate for 60 min. During this period, tissues were washed every 15 min. The endothelium was considered to be present (E+) when the Ach-induced relaxation was at least 80% after pre-contraction with Phe (10−6 M). Endothelium was deemed to be absent (E−) when the relaxation response did not occur. Next, each ring was washed and re-equilibrated for 30 min. The aortic rings were pre-contracted with Phe (10−6 M) after a stable plateau was reached, and dose-response curves of Sclareol were obtained. The concentration-response assays in the organ baths were carried out in the presence or absence: L-NAME (10−4 M), a nonspecific nitric oxide synthase inhibitor and ODQ (10−4 M), a guanylyl cyclase inhibitor. The preparations were incubated with the inhibitors for 30 min.

### Indirect plasma measurements of NO

Blood samples were collected through the femoral vein after sclareol administration and placed into tubes containing heparin. After blood centrifugation (3000 × g, 10 minutes, 4°C), plasma aliquots were immediately immersed in liquid nitrogen and stored at -70°C until nitrite and nitrate (NOx) measurements. Samples were analyzed in duplicates for NOx using an ozone-based chemiluminescence assay. Briefly, the plasma samples were treated with cold ethanol (1 volume of plasma: 2 volumes of ethanol for 30 minutes at −20°C) and centrifuged (4000 × g, 10 minutes). The NOx levels were measured by injecting 25 *µ*L of the supernatant in a glass purge vessel containing 0.8% of vanadium (III) in HCl (1 N) at 90°C, which reduces NOx to NO gas. A nitrogen stream was bubbled through the purge vessel containing vanadium (III), then through NaOH (1 N), and then into an NO analyzer (Sievers® Nitric Oxide Analyzer 280, GE Analytical Instruments, Boulder, CO, USA).

### Statistical analysis

The data are expressed as means ± the standard error of the mean (SEM). We performed statistical analyzes with two-way repeated-measures analysis of variance (ANOVA) and the Bonferroni post-test, or test t Student was carried out to detect possible differences between the values in the study. P < 0.05 was considered significant. (Prism 5.0, GraphPad Software, San Diego, CA, USA). A sample size of (N = 5) animals per group provided 95% power with a 0.05 significance level to detect a relative 10% reduction in the maximal contraction in pre-contracted vessels and a sample size of (N = 7) animals per group provided 95% power with a 0.05 significance level in protocols of in vivo blood pressure measurement. The number of animals was chosen based on literature.

## Results

**Figure 2:**
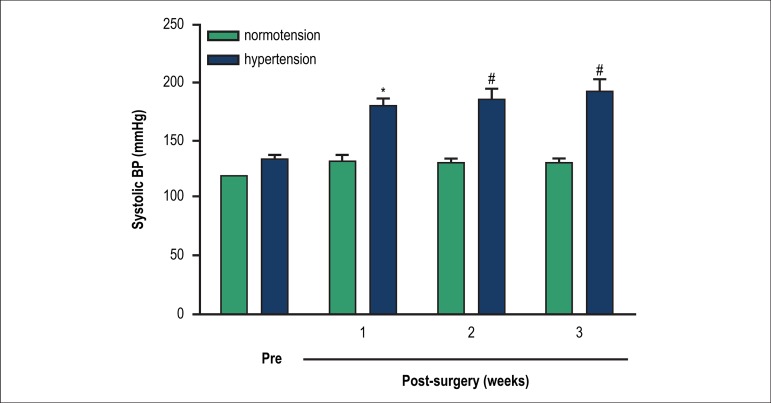
Temporal evolution of noninvasive systolic blood pressure in normotensive and hypertensive animals. The values represent the mean ± standard error of the mean (n = 7). SBP 2K1C before surgery (pre-operative) and at the 3 weeks following surgery. * (p < 0.01) and # (p < 0.001) indicated signifcant difference in the hypertensive group compared to the normotensive group.

Before surgical procedures, there were no differences in the SBP between normotensive and hypertensive groups (sham: 120.7 ± 3.5 mmHg versus 2K1C: 133.8 ± 3.6 mmHg, p > 0.05). However, from the 1^st^ to 3^th^ week after the hypertension induction, the SBP significantly increased in the hypertensive rats (sham at 3^th^ week after surgical procedures: 130.6 ± 3.8 mmHg versus 2K1C group at 3^th^ week after surgical procedures: 192.9 ± 10.2 mmHg, p < 0.001) (Figure 2).

**Figure 3:**
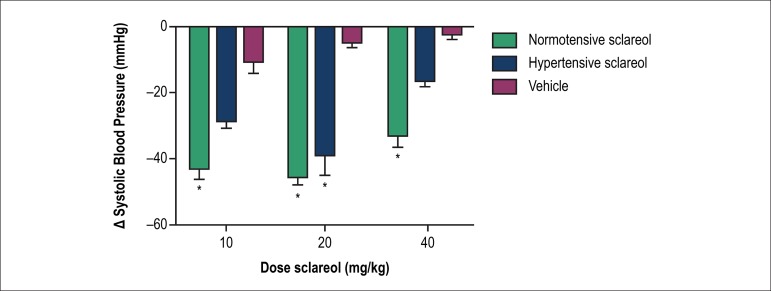
Representative image of the SBP variation, after three doses of Sclareol or vehicle. (n = 7). * (p < 0.01) between vehicle and normotensive or hypertensive groups.

All the three doses of Sclareol (10, 20 and 40 mg/kg) significantly decreased the SBP in the normotensive rats (vehicle: -10.7 ± 6.7 mmHg versus normotensive sclareol: -43.1 ± 7.1 mmHg at 10 mg/kg, p < 0.01; vehicle: -4.8 ± 2.8 mmHg versus normotensive sclareol: -45.5 ± 6.0 mmHg at 20 mg/kg, p < 0.01; vehicle: -2.8 ± 2.3 mmHg versus normotensive Sclareol: -33.3 ± 7.0 mmHg at 40 mg/kg, p < 0.01). Nevertheless, only 20 mg/Kg dose of sclareol change the SBP in the hypertensive animals (vehicle: -4.8 ± 2.8 mmHg versus hypertensive sclareol: -39.1 ± 15.8 mmHg at, p > 0.05) (Figure 3).

**Figure 4:**
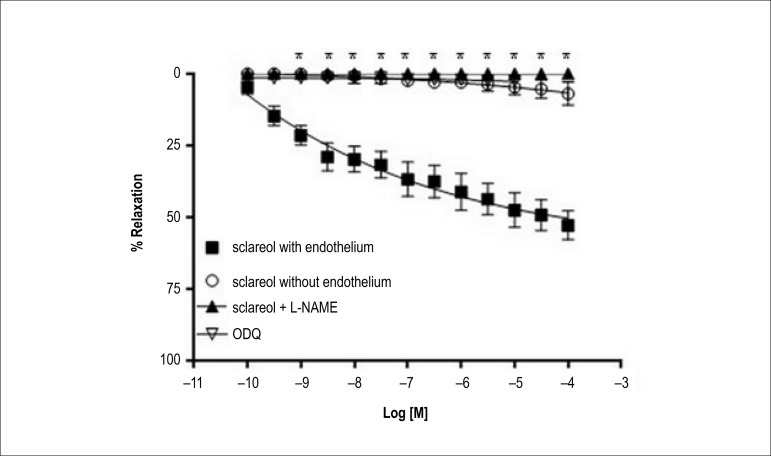
Dose response curve Sclareol in the presence of inhibitors. After the pre- contraction with 10^-7^M Phe, the rings were subjected to a dose response curve from 10^-10^ to 10^-4^ in the presence of L-NAME and inhibitor ODQ. * (p < 0.001) indicate a signifcant difference between the groups with inhibitors and control. (n = 6).

**Figure 5:**
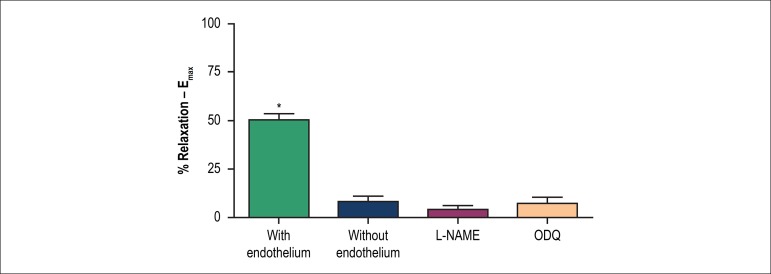
Maximum relaxing effect in the presence of inhibitors. The E_max_ was obtained from dose-response curves, using non-linear regression. * (p < 0.001).

In the case of Phe pre-contracted arteries, Sclareol promoted a dose-dependent relaxation only in intact rings (E+ 52.9 ± 5.0 % versus E- 6,9 ± 4.0%). Incubation with either L-NAME or ODQ totally blocked the relaxation induced by Sclareol in both endothelium-intact rings (Figures 4 and 5).

**Figure 6:**
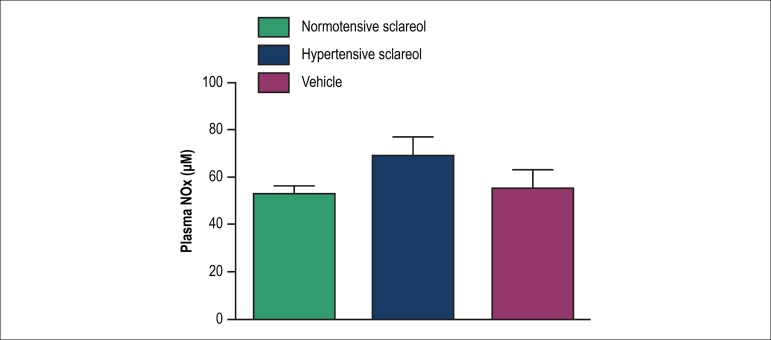
Plasmatic nitrite and nitrate levels (NOx) in normotensive and hypertensive animals. The animals were pretreated with vehicle or Sclareol. (N = 7).

The plasma NOx concentration did not change between all groups group (vehicle: 55.4 ± 7.4 *µ*M; normotensive sclareol: 52.5 ± 3.9 *µ*M and hypertensive vehicle: 68.7 ± 8.3 *µ*M). (Figure 6).

## Discussion

The in vivo results obtained after administration of three escalating doses of Sclareol demonstrate that it promoted a reduction in BP, both in the normotensive and hypertensive animals. The mechanisms involved in this relaxing effect remain unknown. Nevertheless, this effect may be connected with the fact that these compounds are possibly responsible for activation of NO pathways. Looking more deeply into the data collected in 2K1C model, the renin-angiotensin-aldosterone system (RAAS) is the primary factor in the development of hypertension.^13^ In hypertension, there is an activation of the RAAS and in turn, a greater inhibition of kallikrein-kinin system (CMS) by ACE,^14,15^ this can result in a smaller reduction in SBP induced by the Sclareol in the hypertensive group. The largest reduction in SBP in the normotensive group, in response to administration of Sclareol, may be indicative of an interaction between the RAAS and the SCC.^14,15^ However, it takes more experiments to determine the actual cause.

The second mechanism possible involved in the hypotensive effect of sclareol, is the vasodilator property. We tested the Sclareol vasorelaxant effect, in vitro, using isolated rat aortic rings pre-contracted with phenylephrine. The relaxant effect observed from sclareol dose-response curve, in rat aorta denuded-rings, was completely blocked by incubation with L-NAME (non-selective NOS inhibitor) and ODQ (inhibitor of guanylate cyclase), which indicates that Sclareol promotes vasorelaxation via NO/cGMP pathway.

In the present study, indirect plasma measurements of NO were carried out by determination of serum levels of nitrite and nitrate using the SieversNOAnalizer 280i. There were no significant differences between the group treated with Sclareol and the vehicle group. However, the analysis of NO in plasma can be influenced in different stages of the process, because it is a very fine analysis. From this result, the ideal would be measured in real time of NO in isolated endothelial cells stimulated with the compounds. This protocol has been tested in different ways, but we were unsuccessful. After several attempts, we believe that the compounds, in any way interfere with the reading of sly (DAF) used.

## Conclusion

The diterpene Sclareol showed in vivo hypotensive and in-vitro vasodilator effects;

2).The chemiluminescence analysis of the plasmatic NO showed no significant difference between groups and
3) For unknown reasons, the Sclareol is not selective in hypertensive animals. So it is important that further research involving the diterpene Sclareol in the cardiovascular function can be explore more detail about mechanisms of action. From the data obtained in this study, it is concluded that the diterpenes metabolites class would be a promising source prototype for the development of new agents in the cardiovascular therapy.

## References

[1] Rieder C, Strauss G, Fuchs G, Arigoni D, Bacher A, Eisenreich W (1998). Biosynthesis of the diterpene verrucosan-2beta-ol in the
phototrophic eubacterium Chloroflexus aurantiacus. A retrobiosynthetic NMR
study. J Biol Chem.

[2] Barnes PJ, Dweik RA, Gelb AF, Gibson PG, George SC, Grasemann H (2010). Exhaled nitric oxide in pulmonary diseases: a comprehensive
review. Chest.

[3] Ricciardolo FL, Sterk PJ, Gaston B, Folkerts G (2004). Nitric oxide in health and disease of the respiratory
system. Physiol Rev.

[4] Shaul PW (2002). Regulation of endothelial nitric oxide synthase location,
location, location. Annu Rev Physiol.

[5] Caissard JC, Olivier T, Delbecque C, Palle S, Garry PP, Audran A (2012). Extracellular localization of the diterpene sclareol in clary
sage (Salvia sclarea L , Lamiaceae). PLoS One.

[6] Ulubelen A, Topcu G, Eriş C, Sönmez U, Kartal M, Kurucu S (1994). Terpenoids from Salvia sclarea. Phytochemistry.

[7] Huang GJ, Pan CH, Wu CH (2012). Esclareol exhibits anti-inflammatory activity in both
lipopolysaccharide-stimulated macrophages and the
λ-carrageenan-induced paw edema model. J Nat Prod.

[8] Noori S, Hassan ZM, Mohammadi M, Habibi Z, Sohrabi N, Bayanolhagh S (2010). Esclareol modulates the Treg intra-tumoral infiltrated cell and
inhibits tumor growth in vivo. Cell Immunol.

[9] Dimas K, Kokkinopoulos D, Demetzos C, Vaos B, Marselos M, Malamas M (1999). The effect of Esclareol on growth and cell cycle progression of
human leukemic cell lines. Leuk Res.

[10] Mahaira LG, Tsimplouli C, Sakellaridis N, Alevizopoulos K, Demetzos C, Han Z (2011). The labdane diterpene sclareol (labd-14-ene-8, 13-diol) induces
apoptosis in human tumor cell lines and suppression of tumor growth in vivo
via a p53-independent mechanism of action. Eur J Pharmacol.

[11] Golsblatt H, Lynch J, Hangal RF, Summerville WW (1934). Studies on experimental hypertension: I. The production of
persistent elevation of systolic blood pressure by means of renal
ischemia. J Exp Med.

[12] Measuring mouse & rat blood pressure.

[13] Ferrario C, Carretero O, de Jong W (1984). Hemodynamics of experimental renal hypertension. Handbook of hypertension: experimental and genetic models of
hypertension.

[14] Martinez-Maldonado M (1991). Pathophysiology of renovascular hypertension. Hypertension.

[15] Navar LG, Zou L, Von Thun A, Tarng Wang C, Imig JD, Mitchell KD (1998). Unraveling the mystery of goldblatt hypertension. News Physiol Sci.

